# The Bacillus Influenzæ

**Published:** 1910-12-03

**Authors:** 


### Bacteriology.

### THE BACILLUS INFLUENZÆ.

The diagnosis of influenza is made too easily; it should be checked bacteriologically in any case in which an accurate diagnosis is of real importance—phthisis and other maladies are too apt to be mislabelled influenza in their early stages. The difficulty is that modern researches all carry one in the direction of increasing complexity. Nothing medical that is fully examined into proves so simple as it may seem to be at first sight. The following are the carefully considered conclusions of Dr. Thursfield in regard to influenza bacilli.

He holds that there is a general consensus of opinion that the organism originally described by Pfeiffer as the bacillus influenzæ is really the cause of epidemic influenza; that in epidemic influenza it is rarely found in the blood except as an agonal phenomenon; that, nevertheless, in certain cases of endocarditis and of septicæmia an organism identical in all respects with bacillus influenzæ can be isolated, and that it is in all probability the cause of these more serious degrees of illness; that organisms identical in all respects with the bacillus influenzæ are found in a number of patients who die of the acute specific fevers, especially in the broncho-pneumonic areas; that Bordet's bacillus, an organism identical in shape and size with the bacillus influenzæ but capable of differentiation in culture, is the probable cause of pertussis; that a bacillus identical with bacillus influenzæ both in morphological and cultural characteristics, but capable of differentiation by a study of its pathogenic effects upon animals, is the cause of a septicæmic form of cerebro-spinal meningitis; that an organism identical in all respects—morphological, cultural, and pathogenic—with bacillus influenzæ is a cause of suppuration in the middle ear and in the sinuses of the nose; and, finally, that a consideration of the foregoing proportions renders it certain that we must in future recognise that the organisms hitherto described as bacillus influenzæ are not all identical with it, but, like the streptococci, staphylococci, and the coli-typhoid family, belong to a group the various members of which possess very different pathogenic powers.

